# Acute mesenteric ischemia: guidelines of the World Society of Emergency Surgery: a brief radiological commentary

**DOI:** 10.1186/s13017-018-0197-y

**Published:** 2018-07-27

**Authors:** Maria Antonietta Mazzei

**Affiliations:** Department of Medical, Surgical and Neuro Sciences, Diagnostic Imaging, University of Siena, Azienda Ospedaliera Universitaria Senese, 53100 Siena, Italy

**Keywords:** Mesenteric ischemia, CT, Non-occlusive mesenteric ischemia

## Abstract

The aim of this letter is to report some deeper explanations regarding the role of imaging in acute mesenteric ischemia.

The correlation between some computed tomography findings and the cause of ischemia as well as other main factors that could condition the computed tomography appearance of this critical issue is also discussed.

## Dear Editor,

I have read with great interest your article entitled “*Acute mesenteric ischemia: guidelines of the World Society of Emergency Surgery*” by Miklosh Bala et al. [1].

I totally agree with the authors that computed tomography angiography (CTA) has supplanted formal angiography as the diagnostic study of choice in acute mesenteric ischemia (AMI) and on the recommendation that it should be performed as soon as possible for any patient with clinical suspicion of AMI, because it could reduce the delay in diagnosing this critical condition, especially in non-occlusive forms (non-occlusive mesenteric ischemia, NOMI) [2]. CTA, in particular, allows to differentiate occlusive from non-occlusive forms and to evaluate the state of the bowel walls, dictating the appropriate treatment planning (revascularization vs non-vascular management).

Furthermore, I completely support their assertion regarding the fact that in AMI, CTA often requires skilled radiologists to interpret the findings, especially for an early diagnosis in non-occlusive form, even if NOMI requires a high index of suspicion based on a patient’s physiology even without CT.

At this point, I would like to highlight that thanks to some experimental studies [3–5], the accuracy of imaging on this topic has improved a lot in recent years; therefore, from a radiological point of view, some deeper explanations are necessary.

First of all, CTA features of AMI (both vascular and non-vascular findings, such as bowel and mesentery findings) depend on the cause of ischemia: for example, among the occlusive forms of ischemia, in arterial one and venous one, there are highly different features at CTA examination, whereas the CTA features of NOMI appear more similar to those ones of occlusive arterial type. In occlusive acute arterial ischemia and in NOMI, CTA features also result to be similar when a reperfusion process takes place. To make things more complicated, CTA features of AMI also strongly depend on the lapse of time passed from the ischemic event, the efficiency of collateral vessels and finally the presence or absence of a reperfusion process, therefore a re-establishment of normal mesenteric blood supply after the ischemic event.

On the basis of what has been said above, some CTA features generically ascribed to AMI in literature, such as thickening of the bowel wall, and in particular of the small bowel wall, or mesenteric fat stranding, could have a different meaning depending on the different type of AMI, the time passed between the ischemic event and CT examination and finally the presence/absence of a reperfusion process [6, 7].

Take for example the thickening of the small bowel wall, which is usually considered in literature as a CTA sign of irreversible ischemia, as reported also in this article, it is definitely a characteristic feature in occlusive venous ischemia because of the venous congestion (since in the early phase of this type of ischemia), whereas it could be found in occlusive arterial ischemia or in NOMI only if a reperfusion process is present, but never in absence of reperfusion. Indeed, in the advanced phase of these two types of ischemia (arterial occlusive type and NOMI), if reperfusion does not take place, the bowel walls remain thin because there is not any blood flow inside them [8, 9].

Secondly, CTA examination is remarkably important in the management of AMI since it lets evaluate non vascular findings, such as bowel, mesenteric and peritoneal findings, that can change over time suggesting the evolution of this dynamic condition.

>For example, mesenteric fat stranding, which is a typical finding in venous occlusive ischemia (since from the early phase due to the congestion of mesenteric veins caused by the vein occlusion), appears in arterial occlusive type of ischemia and in NOMI only in presence of reperfusion process and it gets worse if the reperfusion process turns out to be ineffective while it decreases or becomes less evident if the reperfusion process turns out to be effective [10].

Therefore, through the analysis of extra-vascular findings, CTA shows a pivotal role in monitoring the evolution of AMI, especially in non-occlusive forms where a medical treatment should be preferred, while surgery should occur only in case of bowel wall necrosis which can be clearly demonstrated and topographically mapped by CTA [11].

The main CT findings of AMI, according to the cause (occlusive, arterial or venous, and non-occlusive type of ischemia), the time passed from the ischemic event and to the presence/absence of reperfusion are schematized in Table 1.

**Table 1:** CT findings in AMI, according to the cause, the time passed from the ischemic event and the presence/absence of reperfusion process

CT findings	AVI	AAI without reperfusion	NOMI without reperfusion	AAI with reperfusion	NOMI with reperfusion
Vessel	Occlusion	Occlusion	No occlusion	Partial occlusion	No occlusion
Mesentery	Fat stranding	Pale mesentery	Pale mesentery	Fat stranding	Fat stranding
	Mesenteric fluid	Fat stranding, mesenteric fluid and pneumatosis in late phase	Fat stranding, mesenteric fluid and pneumatosis in late phase	Mesenteric fluid	Mesenteric fluid
Small bowel	Thickening of the wall (due to vascular congestion and submucosal edema)	Paper-thin appearance of the wall Pneumatosis and absence of contrast enhancement in late phase	Paper-thin appearance of the wall Pneumatosis and absence of contrast enhancement in late phase	Thickening of the wall (due to increased permeability of damaged vessels)	Thickening of the wall (due to increased permeability of damaged vessels)
Peritoneal cavity	Free peritoneal fluid in late phase	Free peritoneal fluid or air in late phase	Free peritoneal fluid or air in late phase	Free peritoneal fluid since the early phase of reperfusion; the increase of free peritoneal fluid is a sign of worsening	Free peritoneal fluid since the early phase of reperfusion; the increase of free peritoneal fluid is a sign of worsening

*AVI* acute venous ischemia, *AAI* acute arterial ischemia, *NOMI* non-occlusive mesenteric ischemia

From a technical point of view, CTA should be performed both with pre-contrast and post-contrast scans (arterial and venous). In particular pre-contrast scan is useful to detect a hyperattenuation of the bowel wall which could mean intramural haemorrhage but also a reperfusion process. A precise diagnosis of these two conditions (intramural haemorrhage or reperfusion process) can be done in post-contrast scan by the evaluation of the increasing of Hounsfield unit (HU) values, which occurs only in the case of reperfusion. Conversely, in case of intramural haemorrhage, the hyperattenuation of the bowel wall does not change its HU values in post-contrast CT scan, meaning the absence of contrast enhancement of the bowel wall.

As far as concerns ultrasound (US), it has certainly a limited role in this critical condition; however, it could be useful to monitor the peristalsis of the bowels or the amount of free peritoneal fluid especially in NOMI under medical control [12].

Finally, even magnetic resonance imaging (MRI) could have a remarkable role in AMI, especially in NOMI to monitor an effective reperfusion, because it can be performed without contrast media administration and avoiding dose exposure to radiations during patient’s close checks [13].

As researcher in the field of AMI, I have come to the conclusion that imaging has a remarkable importance and despite its complexity it allows early diagnosis, much better than in the past, provided it is correctly performed and very well interpreted.

## Abbreviations

AMI: Acute mesenteric ischemia
CTA: Computed tomography angiography
HU: Hounsfield unit
MRI: Magnetic resonance imaging
NOMI: Non-occlusive mesenteric ischemia
US: Ultrasound
